# Sequence Coevolution between RNA and Protein Characterized by Mutual Information between Residue Triplets

**DOI:** 10.1371/journal.pone.0030022

**Published:** 2012-01-18

**Authors:** Relly Brandman, Yigal Brandman, Vijay S. Pande

**Affiliations:** 1 Chemical and Systems Biology, Stanford University, Stanford, California, United States of America; 2 Chemistry, Stanford University, Stanford, California, United States of America; University of Helsinki, Finland

## Abstract

Coevolving residues in a multiple sequence alignment provide evolutionary clues of biophysical interactions in 3D structure. Despite a rich literature describing amino acid coevolution within or between proteins and nucleic acid coevolution within RNA, to date there has been no direct evidence of coevolution between protein and RNA. The ribosome, a structurally conserved macromolecular machine composed of over 50 interacting protein and RNA chains, provides a natural example of RNA/protein interactions that likely coevolved. We provide the first direct evidence of RNA/protein coevolution by characterizing the mutual information in residue triplets from a multiple sequence alignment of ribosomal protein L22 and neighboring 23S RNA. We define residue triplets as three positions in the multiple sequence alignment, where one position is from the 23S RNA and two positions are from the L22 protein. We show that residue triplets with high mutual information are more likely than residue doublets to be proximal in 3D space. Some high mutual information residue triplets cluster in a connected series across the L22 protein structure, similar to patterns seen in protein coevolution. We also describe RNA nucleotides for which switching from one nucleotide to another (or between purines and pyrimidines) results in a change in amino acid distribution for proximal amino acid positions. Multiple crystal structures for evolutionarily distinct ribosome species can provide structural evidence for these differences. For one residue triplet, a pyrimidine in one species is a purine in another, and RNA/protein hydrogen bonds are present in one species but not the other. The results provide the first direct evidence of RNA/protein coevolution by using higher order mutual information, suggesting that biophysical constraints on interacting RNA and protein chains are indeed a driving force in their evolution.

## Introduction

A primary cause of coevolution between residues is biophysical interactions in the corresponding folded structure. Commonly a sequence slowly changes over evolution while the native fold is maintained, and coevolving positions have been observed in residues proximal in the 3D structure [1], [2], [3], [4], [5], [6], [7], [8], suggesting a link between coevolving positions and structure. Coevolving positions between residues far apart in protein structure [6], [7] and even in different genes [3] have also been observed, hinting at the complexities of the evolutionary process. In addition, phylogenetic influences and small sample size can decrease the signal to noise, making it difficult to identify coevolving positions [5], [9]. Nevertheless, coevolving positions appear to be enriched in areas that are nearby in 3D space and characterizing coevolution is of fundamental importance.

Mutual information (MI) is one of a handful of different methods used to characterize coevolution and has been successful in identifying coevolving pairs of positions in multiple sequence alignments (MSAs) [5], [7], [9], [10]. In an MSA, MI directly measures the dependence of one position in the sequence on another. Previous studies have used MI towards uses such as residue contact prediction in proteins [5] and RNA structure prediction [10]. Multiple pairs of coevolving amino acids that form a connected structure across a protein have been characterized and implicated in functional roles such as interaction surfaces [7]. Folded proteins and RNAs are dense, highly connected structures, and undoubtedly have higher-order biophysical interactions. Triplet structures formed by hydrogen bonding patterns between three RNA nucleotides [11] and between an amino acid and RNA base pair side chains [12] have been previously described. There are likely higher order patterns in coevolution.

Due to the biophysical interactions between RNA and protein present in joint complexes, it is natural to hypothesize that protein and RNA have coevolved in these complexes. However, to date no study has been able to demonstrate RNA/protein coevolution in the sequence record. Due to the wealth of sequence and structural data, the ribosome (a large macromolecular complex made of multiple RNA and protein chains [13], [14], [15], [16] that catalyzes protein synthesis in all living cells) is a natural test bed to examine RNA/protein coevolution. Ribosome structure is remarkably conserved over the four known bacteria and archeon species with crystal structures [13], [14], [15], [16], hinting at the importance of structure in ribosome function. A detailed study of a ribosome crystal structure revealed a handful of RNA/protein interactions that can be characterized by features such as hydrogen bonding, with most interactions between protein side chains and the RNA backbone [15]. Many of the residues catalogued in this study are conserved over evolution however (for example, all of the interactions between protein L22 and RNA involve conserved residues), and therefore by definition do not coevolve. Non-conserved residues may also interact, and MSAs of ribosome chains provide a rich dataset to begin to characterize RNA/protein coevolution, one that complements structural information from crystal structures.

Here, we describe the coevolution between RNA and protein in the ribosome by calculating the MI between triplets of positions in an MSA. We define residue triplets as three positions in the multiple sequence alignment, where one position is from the 23S RNA and two positions are from the L22 protein. Using higher order coevolution patterns reveals an increased likelihood for residues proximal in 3D space to coevolve, whereas coevolution between pairs of positions does not show this pattern. Triplets of RNA with polar amino acids show even higher coevolution at close 3D distance, probably related to the increased likelihood of polar amino acids for being on the surface of the protein and for interacting with RNA because of RNA's negative charge. Our dataset focuses on residues from a protein in the large subunit, L22, and the RNA within 10 Å of L22 and we calculate the MI between triplets of positions.

Mutual information between three variables, MI(X, Y, Z), is more complex than mutual information between two variables, MI(X,Y). MI(X,Y) measures the dependence between two variables. Two dependent variables have high MI(X,Y), and two independent variables have MI(X,Y) of zero. M(X,Y,Z) measures the interaction between three variables. MI between three variables can be defined in the following way:
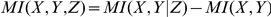
Thus MI(X,Y,Z) is the difference between the mutual information between X and Y when Z is fixed and when Z is not fixed. MI is symmetric, thus MI(X,Y) = MI(Y,X) and MI(X,Y,Z) = MI(Y,X,Z) = MI(Z,Y,X) = MI(Z,X,Y) = MI(X,Z,Y) = MI(Y,Z,X). The upper bound of MI is the (Shannon [17]) joint entropy (H(X,Y) for MI(X,Y) and H(X,Y,Z) for MI(X,Y,Z). For our data set, the variables are positions in an MSA. High mutual information between two or three positions in the MSA indicates that these residues coevolved, and low mutual information indicates that the positions evolved independently. Entropy in a position in an MSA indicates the uncertainty, in other words the variability in the amino acid residue.

Why study this system? The functions of ribosomal proteins are poorly understood. Ribosomal proteins may play a role during ribosome assembly, contribute to structural stability, and/or aid in translation. Nascent chains translated by the ribosome encounter a beta hairpin in L22 immediately after the formation of a new peptide bond at the catalytic site, and L22 likely plays a regulatory role in this process [18]. L22 makes contact with all six domains of the largest RNA chain in the ribosome, the 23S, and is also necessary for forming an early 23S folding intermediate [19]. Thus L22 also plays a role in ribosome assembly. L22 was chosen for this study because of its small size relative to other ribosome proteins (therefore less computationally demanding) and functional importance. Focusing on these particular residues in the ribosome provides a starting point for characterizing RNA/protein coevolution.

Increased coevolution between RNA/protein triplets at close 3D space suggests the influence of RNA/protein biophysical interactions in evolution, and we use our data in conjunction with ribosome crystal structures to gain insight into this process. For two RNA nucleotides, their respective high MI triplets cluster in a series connected across the structure, similar to patterns seen in protein coevolution [7], [8]. We describe a typical residue distribution pattern for high MI, proximal triplets in which switching from one nucleotide to another (or between purines and pyrimidines) results in a distinct change in the pattern of nearby amino acids. One type of pattern leading to high MI is an amino acid distribution that is narrow for certain RNA values (i.e. for a given RNA nucleotide in position Z, there are only a few amino acids in positions X and Y), and otherwise wide. We highlight a specific triplet, U519/R18/D22, in which a pyrimidine is most likely accompanied by an Arg (R) and Asp (D) and a purine accompanied by a more varied distribution of amino acids. A structural alignment of the crystal structures of two species, one with a pyrimidine and the other a purine at position U519, reveals a hydrogen bonding pattern that is broken upon mutation. A goal of this study is to connect coevolution and biophysical interactions by looking for increased coevolution at close 3D distance, and this is the first study to do so for RNA and protein.

## Results

MI was calculated between one nucleotide and two amino acids from bacterial MSAs for the L22 protein and the large subunit RNA chain that binds to the L22 protein, the 23S (see Methods for details). Conserved residues by definition do not coevolve, and we exclude these residues with the use of an entropy cutoff of 0.3, a commonly used threshold for pairwise MI [5]. In our manuscript, entropy is considered to be the Shannon entropy [17] in the amino acids in certain positions in the MSA. There are 75 nucleotides within 10 Å of L22, and 39 of these are conserved. There are 113 residues in the L22 protein, and 8 of these are conserved. We also exclude the three c-terminal amino acids from the analysis because their position makes them unlikely to interact with RNA and their high entropy results in noisy MI. Normalizing MI has been shown to increase residue contact prediction, and an effective method to normalize MI is to report the ratio of MI to the entropy (MI/H) [20]. This normalized form of MI will be used throughout (see Methods for details).

### RNA/protein triplet coevolution is enhanced at close 3D distance

**Figure 1:**
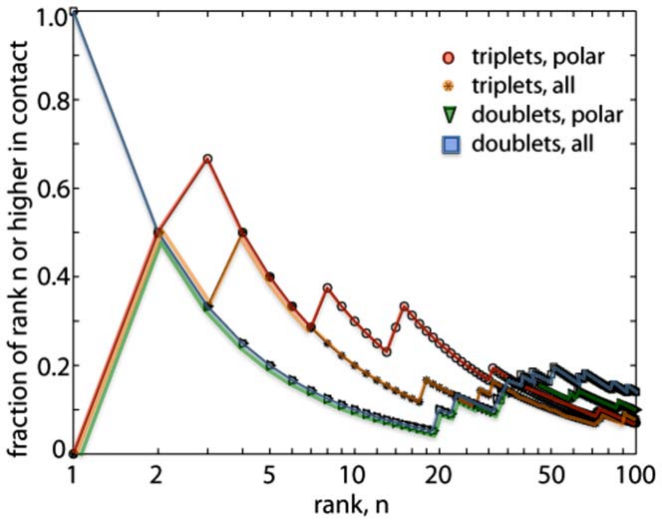
Likelihood of contact between triplets (orange *, red o) and pairs (blue □, green ∇) of residue positions vs. the coevolution rank. High MI triplets are likely to be in contact for triplets of residue positions (similar to coevolution seen in protein position pairs [5]), but doublets are not. For example, 40% of the top 5 highest ranking MI triplets are in contact, while only 20% of the top 5 highest ranking MI doublets are in contact. MI between RNA and polar amino acids, more likely to lie on the surface on the protein and therefore interact with RNA, enhances the trend (o triplets, ∇ doublets). High MI triplets between polar amino acids and RNA are most likely to be in contact. In comparison, random coevolution between pairs of amino acids is expected to have a contact frequency of 8% [5].

A common metric for reporting MI data is to rank the highest MI pairs of residues (and in our case, triplets) and classify them as “in contact” if their 3D coordinates are within a distance threshold [5], and we report the top 100 RNA/protein pairs and triplets from our dataset in Figure 1. Pairs are between positions representing one amino acid in the L22 protein and one nucleotide from an RNA neighbor within 10 Å of L22. Triplets are between positions representing two amino acids in the L22 protein and one nucleotide from an RNA neighbor within 10 Å of L22. For positive MI values, higher MI indicates greater dependence between the positions in the MSA, and hence greater coevolution between the positions. MI between two variables is always non-negative. MI between three variables can be negative, zero, or positive. In our dataset, the triplets with negative MI values largely involve amino acids that evolve relatively independently, and here we only characterize positive MI values to simplify the analysis (see Discussion for details). Our distances are between backbone atoms, and we classify residues < = 12 Å as “in contact” (see Methods for details).

High MI RNA/protein triplets are likely to be close in 3D space (Figure 1, asterisks), whereas high MI RNA/protein doublets do not show this pattern (Figure 1, squares). Likelihood of contact for RNA/protein triplets is on the same order as what was previously described for amino acids doublets [5]. Polar amino acids are more likely to interact with RNA because they are more likely to be on the surface of the protein and more likely to form electrostatic interactions with the negatively charged RNA nucleotides, and we see an increased likelihood for contact in RNA/protein doublets (Figure 1, circles) and triplets (Figure 1, triangles) with polar amino acids. The following residues were included as polar in our analysis: Asp (D), Glu (E), His (H), Lys (K), Asn (N), Gln (Q), Arg (R), Ser (S), Thr (T), and Tyr (Y). The similarities between our dataset and those previously characterized for proteins, and the increased fraction of high MI triplets in contact when filtering for polar amino acids is suggestive of biophysical interactions between the RNA and protein.

Low signal to noise is a known problem when looking for coevolution in MSAs. In our dataset, there are far fewer triplets at close distances than there are at far distance due to the relative orientations of the nucleotides and amino acids (as well as the general combinatorics of triplets). In addition to differences in sample size at different distance bins, noise can occur because of insufficient number of sequences. It has been shown that at least 125 protein sequences are needed to compensate for background noise in MI [20]. Our MSA has ∼400 sequences from different bacterial species, although the number of species for each individual triplet is less than 400 because we do not incorporate gaps in our calculations (see Methods for more details). We normalize MI values to the entropy, a method that has been shown to increase the signal to noise [20]. In addition to statistical noise, coevolution may arise for reasons other than biophysical interactions (e.g. phylogenetic relationships or functional relationships).

### U519 and C487: Highest MI triplets form a connected series in 3D structure

**Figure 2:**
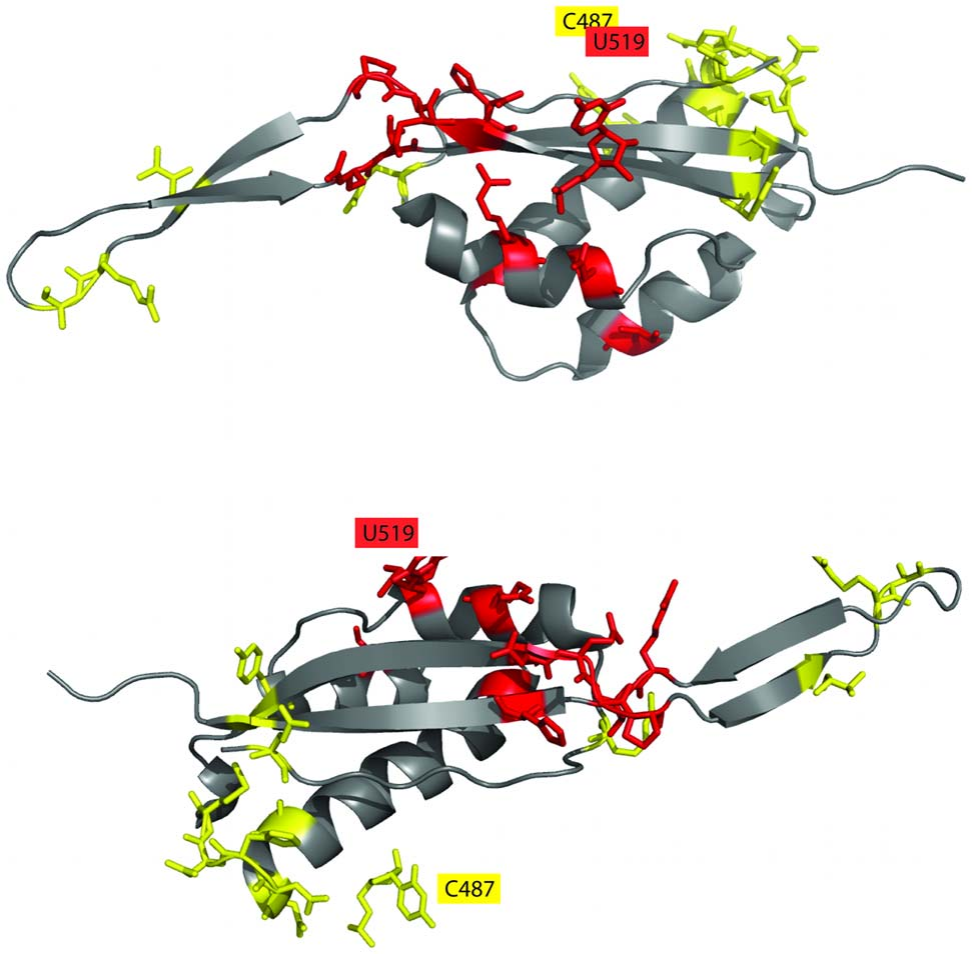
The top ten highest MI triplets for U519 (2A, red) and C487 (2B, yellow) form a connected series across the protein structure (standard *E.coli* numbering). Both U519 and C487 form base pairs with other 23S nucleotides, and thus all interactions are via backbone atoms. Many of the amino acids in the high MI clusters are at the edges of secondary structure elements. Other nucleotides did not have high MI triplets clustered in 3D space.

Figure 2 shows the residues in the top ten highest MI triplets for U519 (Figure 2A) and C487 (Figure 2B), and these residues form a connected series on the protein structure. 23S RNA chain from *E.coli* standard numbering is used throughout. U519 and C487 are both base paired to other residues in the 23S RNA chain, and thus their interaction with L22 is via backbone atoms. L22 has two domains; an extended region that is buried in the large subunit of the ribosome, and a globular domain that is partly solvent exposed. The globular domain has alpha helices and antiparallel beta sheets, and both clusters are in this globular domain. The U529 high MI cluster is at the extended region end of the globular domain, and the C487 high MI cluster is at the solvent exposed end of the globular domain. Many of the amino acids in these clusters are at the edges of secondary structure elements, a structural link that has been previously described in coevolving amino acids [7]. Two classes of coevolving resides in amino acids have been previously characterized, those that coevolve with only a few residues and those that coevolve with a series of residues [7], [8], and we see both classes in our RNA/protein triplets. Triplets with other RNA nucleotides in our dataset had high MI proximal triplets, but their respective top ten highest MI triplets did not cluster in 3D space.

### High MI proximal triplets show shifts in amino acid distributions upon changes in RNA

High MI, proximal triplets have different amino acid distributions with different RNA bases, and a triplet with RNA U519 is shown as an example. U519 is adjacent to helix 1 and beta strand 2 in the L22 protein, and all four nucleotides are represented in our MSA (adenine 5%, cytosine 10%, guanine 15%, and uracil 70%). In the majority of sequences, U519 maintains a base pair irrespective of its value (i.e. if U519 mutates to an G its base pair partner will mutate to a C, data not shown).

**Figure 3:**
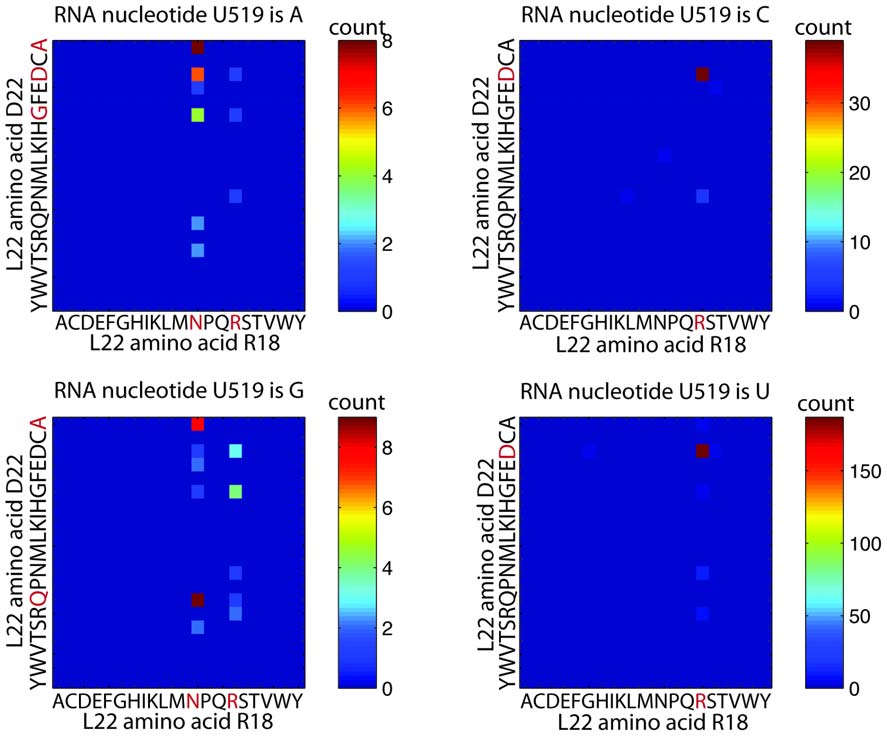
Residue distributions for the most proximal high MI triplet with U519, U519/R18/D22, a typical high MI proximal triplet. A pyrimidine (RNA is C or U) results in a tight distribution in which R18 is an Arg (R) and D22 is an Asp (D). A purine (RNA is A or G), slightly smaller than pyrimidines, results in a more diverse distribution in which R18 is most commonly an Asn (N) or Arg (R) and D22 is more widely distributed.

The most proximal high MI triplet with U519 is U519/R18/D22 and the triplets from the MSA are shown in Figure 3. Each of the four 20×20 matrixes represent the amino acids values at a particular RNA value, and thus give a visual representation of the distributions used in the MI calculation (see Methods for details). The U519/R18/D22 triplet is typical of other high MI proximal triplets in that a change in the RNA shifts the distributions of amino acids. In this triplet, a pyrimidine (RNA is C or U) results in a tight distribution in which R18 is an Arg (R) and D22 is an Asp (D). A purine (RNA is A or G), slightly smaller than pyrimidines, results in a more diverse distribution in which R18 is most commonly an Asn (N) or Arg (R) and D22 is more widely distributed. See Figures S1 and S2 for examples of high MI triplets from RNAs C487, G2009 and C2815.

### High MI proximal triplets show hydrogen bonding patterns in the crystal structure that are broken upon mutation

**Figure 4:**
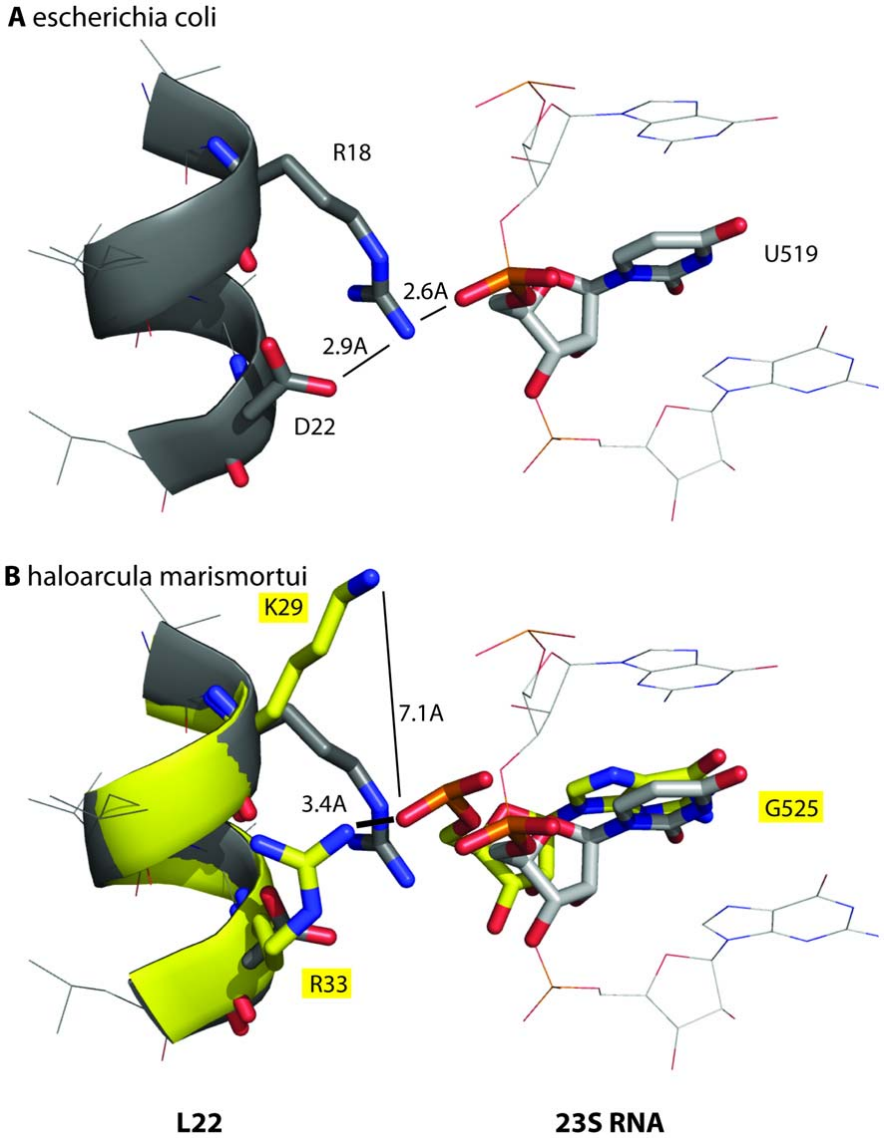
Structural evidence explains the residue distributions for triplet U519/R18/D22. In E.coli, the values of RNA U519/L22 R18/L22 D22 are U, Arg (R) and Asp (D), respectively. A hydrogen bond network in E.coli goes from the side chain of D22 to the side chain of R18 to the phosphate atom of U519. (Figure 4A), and explains the tight coupling seen in the distribution (Figure 3). The triplet from the archeon haloarcula marismortui represents a shift from pyrimidine to purine, with the values of U519/R18/D22 at G, Lys (K) and Arg (R), respectively. A structural alignment of the crystal structure from both species reveals that the hydrogen bonds are broken when the RNA is a purine and the residues farther apart (Figure 4B). This data suggests that the change in packing to accommodate a larger RNA side chain influences the packing between the L22 and 23S protein in such a way that this hydrogen bond network is broken.

The most proximal high MI triplet with U519, U519/R18/D22 described in Figure 3, shows a triplet hydrogen bonding pattern in the *e.coli* crystal structure [13]. In *E.coli*, the values of U519/R18/D22 are U, Arg (R) and Asp (D), respectively. The distance between the three residues is 7.7 Å and MI/H 0.08 (see Methods for details). The side chains of L22 R18 and L22 D22 and the phosphate backbone of RNA U519 are within hydrogen bonding distance (Figure 4A), and a stable structure for this triplet explains the tight coupling seen in the distribution (Figure 3). The triplet from the archeon *haloarcula marismortui* represents a shift from pyrimidine to purine, with the values of U519/R18/D22 at G, Lys (K) and Arg (R), respectively. A structural alignment of the crystal structure from this species [15] with the crystal structure of *E.coli* indicates a structural shift in which the hydrogen bonds are broken and the residues farther apart (Figure 4B). The crystal structures from these two species present a structural argument for the distributions seen in the frequencies, although not all high MI proximal triplets show hydrogen bonding patterns. Our data indicates that RNA side chains can influence the biophysical interactions with proximal amino acids in the following two ways: directly (for example, a hydrogen bond between an amino acid and RNA side chain) and indirectly (for example, by influencing the packing and thus indirectly influencing neighboring interactions).

## Discussion

MSAs represent a rich dataset for studying evolution and elucidating the relationship between linear primary sequences and folded structures continues to be of fundamental importance. Correlations between positions in an MSA, coevolution, has been studied in light of evidence suggesting enhanced coevolution for residues that interact in 3D space [1], [2], [3], [4], [5], [7], [8], [20], although non-proximal residues also coevolve in an undoubtedly complex evolutionary process. This link between sequence and structure has resulted in numerous methods for characterizing coevolution between pairs of positions in an MSA, including information theoretic methods such as MI [5], [7], [20]. Previous studies have characterized coevolution between pairs of positions for either proteins or RNA, but to date there has been no study describing RNA/protein coevolution. RNA/protein interactions are of fundamental importance in biology, including the interactions in the multi-chain macromolecular structure of the ribosome, and thus it is natural to hypothesize that these sequences coevolved. Thus, our result that one can see this coevolution in the MSAs is an important discovery, yielding evidence to support this hypothesis.

To characterize and quantify RNA/protein coevolution we calculated the MI between triplets of positions in an RNA/protein MSA from chains in the ribosome and reveal increased MI at close 3D distances, suggesting the importance of biophysical interactions. We turned to higher dimensional MI because MI between doublets was not enhanced for residues close in 3D distance. This could be because there is less noise in triplet MI calculation, or because RNA forms stable higher dimensional structures with proteins, or both. MI between triplets is mathematically more complex than MI between doublets. An analogy can be made between MI of triplets and 3D distance between three points. 3D distance between two points is unambiguous, whereas there are many ways for three points to be 10 Å apart (e.g. two points close together and one farther away, or three equidistant points). Adding to the complexity is the fact that MI between triplets can be negative, zero, or positive. MI between doublets is either zero or positive. For positive MI, the higher the number the greater the dependence between the variables. The equation for MI between triplets can be written in a few different forms, including the following
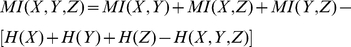
where X, Y, and Z are random variables and H is entropy. The second term in the equation represents the amount of overlap between the entropies of the three variables and is always non-negative. Thus MI is positive when the overlap between the entropies is small relative to the mutual information between pairs of variables, and negative when the mutual information between pairs of variables is small relative to the overlap between the three entropies. In our dataset, most negative MI values probably represent triplets in which the residues evolve relatively independently (low doublet MIs), and for the purposes of this initial study we focus on positive MI values.

We see high MI, proximal RNA/protein triplets as well as two nucleotides with top ranking triplets that form a connected series of residues in the 3D structure, consistent with two types of coevolution previously described in proteins [7]. The likelihood of high MI triplets for being in contact is on the order of what has been previously described for protein doublets [5]. Often a change in nucleotide results in a shift in amino acid distributions. We focus a more in depth study of triplet U519/R18/D22 and show that a shift from pyrimidine to purine results in a shift from a tight Arg (R)/Asp (D) distribution to a more varied distribution of amino acids (Figure 3). We provide a structural interpretation of the data by looking at a structural alignment between two species, one with a pyrimidine and one with a purine in the triplet (Figure 4). In the structure in which the RNA is a pyrimidine, the residues form hydrogen bonds. In the structure in which the RNA is a purine, there is a slight structural shift and the residues are further apart and do not hydrogen bond.

Characterizing sequence covariation is a predictive tool as well as an important methodology to further fundamental understanding of evolution and the relationship between primary and 3D structure, and this is the first study to establish statistical evidence for the coevolution between RNA and protein. There are no simple rules governing RNA/protein interactions and a wide variety of interactions have been observed [12], [15], [21]. Crystal structures suggest that surface complementarity, electrostatic interactions, and hydrogen bonding all play a role and databases of known interactions have been established [12], [21]. Conserved and evolving residues both participate in these interactions. This work details an information theoretic method to establish coevolution between triplets of RNA and protein residues for a functionally important protein in the ribosome. The coevolution patterns seen, namely an increased likelihood for coevolving residues to be proximal and the coevolution of a network of residues across a structure, are similar to the two types of coevolution seen for protein residue pairs [6]. In addition, we characterize residue triplets for which mutations in an RNA nucleotide results in a change in residue distribution for proximal amino acids. Further characterizing RNA/protein coevolution in more systems will increase our fundamental understand of this important process.

## Methods

### Multiple Sequence Alignments

MSA for the 23S was taken from The Comparative RNA Web Site [22] and contains over 6000 sequences. MSA for L22 was created by taking sequences from the NCBI Microbial Genomes Resource (http://www.ncbi.nlm.nih.gov/genomes/MICROBES/microbial_taxtree.html), which had 998 species at time of download, and using clustalw [23] to create an MSA. Of the 998 species in our L22 alignment, 393 overlap with species from the 23S alignment. Because the 23S is approximately 3000 nucleotides long, we chose to use the hand curated alignment at the expense of having less overlapping species with our L22 alignment (as opposed to creating a new alignment from 23S species taken from the NCBI bacterial genome database). Therefore the effective number of species is our MSA is 393. The number of sequences used to calculate MI for each individual pair or triplet may be less than 393 because we do not incorporate gaps into our calculation.

### Mutual Information

MI between three variables is calculated as
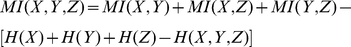
where, MI is mutual information, H is entropy, and X, Y and Z are random variables. In our dataset, the random variables are positions in an MSA. Mutual information between two variables is calculated as
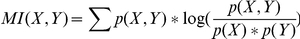
and entropy as
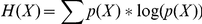
MI is normalized by the joint entropy H(X,Y,Z), and MI/H is reported throughout.

### 3D Distance Between Three Residues

The distance between three residues is calculated as the square of the sum of distances between each point and the center of mass of the three points.

## Supporting Information

Figure S1 **The top ten highest MI triplets for 23S RNA U519 (red) and C487 (yellow), G2009 (green), and C2815 (blue).**
(TIF)

Figure S2 **Residue distributions for the most proximal high MI triplet with G2009 G2009/T39/K42.**
(TIF)

## References

[1] Fitch WM, Markowitz E (1970). An improved method for determining codon variability in a gene and its application to the rate of fixation of mutations in evolution.. Biochem Genet.

[2] Yanofsky C, Horn V, Thorpe D (1964). Protein Structure Relationships Revealed by Mutational Analysis.. Science.

[3] Poon A, Chao L (2005). The rate of compensatory mutation in the DNA bacteriophage phiX174.. Genetics.

[4] Yeang CH, Haussler D (2007). Detecting coevolution in and among protein domains.. PLoS Comput Biol.

[5] Dunn SD, Wahl LM, Gloor GB (2008). Mutual information without the influence of phylogeny or entropy dramatically improves residue contact prediction.. Bioinformatics.

[6] Fares MA, Travers SA (2006). A novel method for detecting intramolecular coevolution: adding a further dimension to selective constraints analyses.. Genetics.

[7] Gloor GB, Martin LC, Wahl LM, Dunn SD (2005). Mutual information in protein multiple sequence alignments reveals two classes of coevolving positions.. Biochemistry.

[8] Lee J, Natarajan M, Nashine VC, Socolich M, Vo T (2008). Surface sites for engineering allosteric control in proteins.. Science.

[9] Wollenberg KR, Atchley WR (2000). Separation of phylogenetic and functional associations in biological sequences by using the parametric bootstrap.. Proc Natl Acad Sci U S A.

[10] Chiu DK, Kolodziejczak T (1991). Inferring consensus structure from nucleic acid sequences.. Comput Appl Biosci.

[11] Levitt M (1969). Detailed molecular model for transfer ribonucleic acid.. Nature.

[12] Cheng AC, Chen WW, Fuhrmann CN, Frankel AD (2003). Recognition of nucleic acid bases and base-pairs by hydrogen bonding to amino acid side-chains.. J Mol Biol.

[13] Schuwirth BS, Borovinskaya MA, Hau CW, Zhang W, Vila-Sanjurjo A (2005). Structures of the bacterial ribosome at 3.5 A resolution.. Science.

[14] Selmer M, Dunham CM, Murphy FVt, Weixlbaumer A, Petry S (2006). Structure of the 70S ribosome complexed with mRNA and tRNA.. Science.

[15] Klein DJ, Moore PB, Steitz TA (2004). The roles of ribosomal proteins in the structure assembly, and evolution of the large ribosomal subunit.. J Mol Biol.

[16] Bashan A, Yonath A (2008). Correlating ribosome function with high-resolution structures.. Trends Microbiol.

[17] Cover TM, Thomas JA (1991). Elements of Information Theory.

[18] Berisio R, Schluenzen F, Harms J, Bashan A, Auerbach T (2003). Structural insight into the role of the ribosomal tunnel in cellular regulation.. Nat Struct Biol.

[19] Unge J, berg A, Al-Kharadaghi S, Nikulin A, Nikonov S (1998). The crystal structure of ribosomal protein L22 from Thermus thermophilus: insights into the mechanism of erythromycin resistance.. Structure.

[20] Martin LC, Gloor GB, Dunn SD, Wahl LM (2005). Using information theory to search for co-evolving residues in proteins.. Bioinformatics.

[21] Morozova N, Allers J, Myers J, Shamoo Y (2006). Protein-RNA interactions: exploring binding patterns with a three-dimensional superposition analysis of high resolution structures.. Bioinformatics.

[22] Cannone JJ, Subramanian S, Schnare MN, Collett JR, D'Souza LM (2002). The comparative RNA web (CRW) site: an online database of comparative sequence and structure information for ribosomal, intron, and other RNAs.. BMC Bioinformatics.

[23] Larkin MA, Blackshields G, Brown NP, Chenna R, McGettigan PA (2007). Clustal W and Clustal X version 2.0.. Bioinformatics.

