# Supplementary figures and images for: Sequence Coevolution between RNA and Protein Characterized by Mutual Information between Residue Triplets
Source: PLoS One. 2012 Jan 18;7(1):e30022. doi: 10.1371/journal.pone.0030022 (PMC3261191; doi:10.1371/journal.pone.0030022)

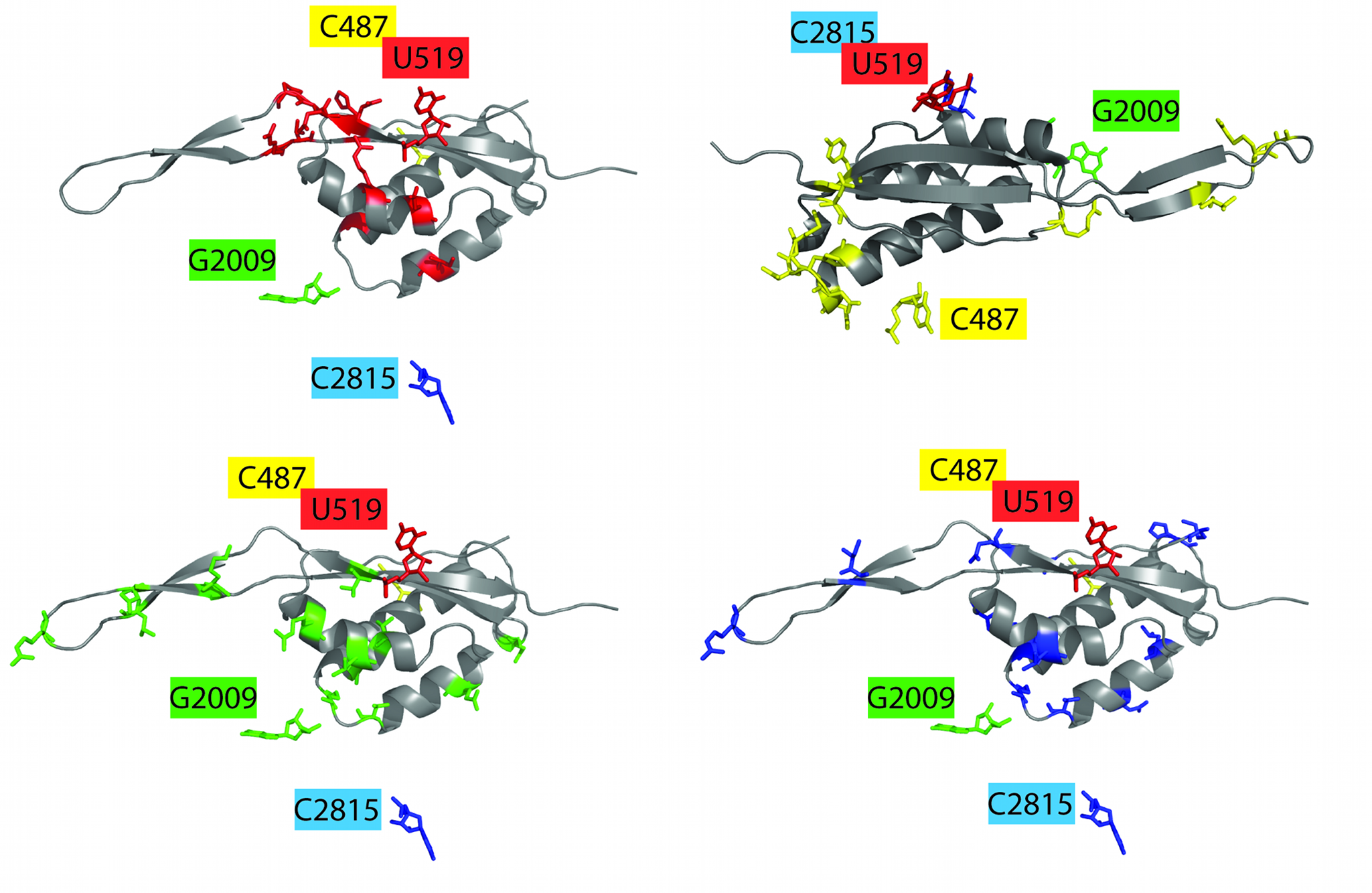

Supplement: Figure S1 — The top ten highest MI triplets for 23S RNA U519 (red) and C487 (yellow), G2009 (green), and C2815 (blue). (TIF) [file pone.0030022.s001.tif]

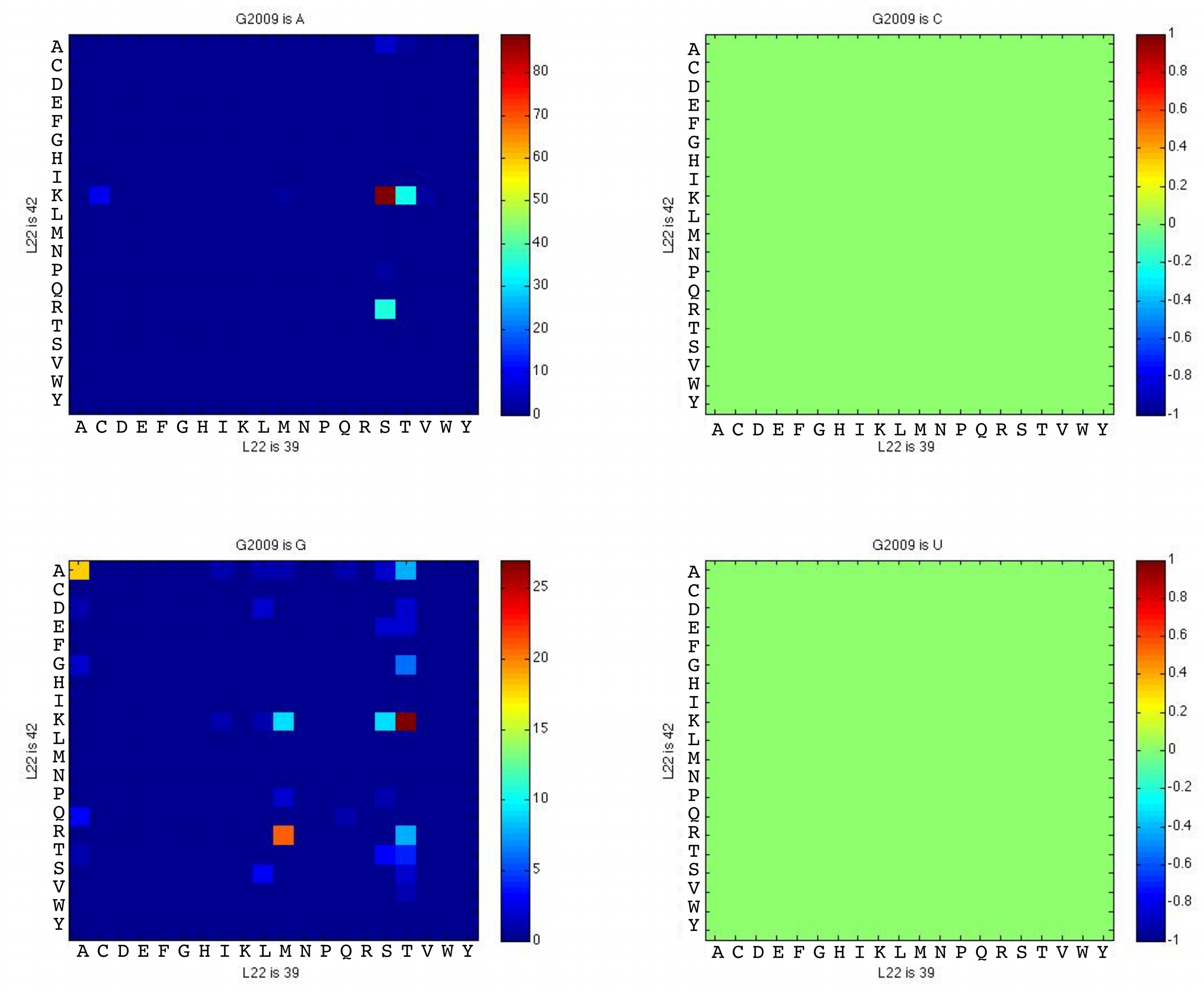

Supplement: Figure S2 — Residue distributions for the most proximal high MI triplet with G2009 G2009/T39/K42. (TIF) [file pone.0030022.s002.tif]
